# Finding minimal action sequences with a simple evaluation of actions

**DOI:** 10.3389/fncom.2014.00151

**Published:** 2014-11-28

**Authors:** Ashvin Shah, Kevin N. Gurney

**Affiliations:** Department of Psychology, The University of SheffieldSheffield, UK

**Keywords:** action discovery, reinforcement learning, intrinsic motivation, optimal control, redundancy, dopamine

## Abstract

Animals are able to discover the minimal number of actions that achieves an outcome (the minimal action sequence). In most accounts of this, actions are associated with a measure of behavior that is higher for actions that lead to the outcome with a shorter action sequence, and learning mechanisms find the actions associated with the highest measure. In this sense, previous accounts focus on more than the simple binary signal of “was the outcome achieved?”; they focus on “how well was the outcome achieved?” However, such mechanisms may not govern all types of behavioral development. In particular, in the process of action discovery (Redgrave and Gurney, 2006), actions are reinforced if they simply lead to a salient outcome because biological reinforcement signals occur too quickly to evaluate the consequences of an action beyond an indication of the outcome's occurrence. Thus, action discovery mechanisms focus on the simple evaluation of “was the outcome achieved?” and not “how well was the outcome achieved?” Notwithstanding this impoverishment of information, can the process of action discovery find the minimal action sequence? We address this question by implementing computational mechanisms, referred to in this paper as no-cost learning rules, in which each action that leads to the outcome is associated with the same measure of behavior. No-cost rules focus on “was the outcome achieved?” and are consistent with action discovery. No-cost rules discover the minimal action sequence in simulated tasks and execute it for a substantial amount of time. Extensive training, however, results in extraneous actions, suggesting that a separate process (which has been proposed in action discovery) must attenuate learning if no-cost rules participate in behavioral development. We describe how no-cost rules develop behavior, what happens when attenuation is disrupted, and relate the new mechanisms to wider computational and biological context.

## 1. Introduction

Animals are capable of executing a huge variety of movements and behaviors, to which we refer collectively as actions. Importantly, animals are able to discover the actions, including sequences of actions, that affect the environment and preferentially recruit them in order to explore the environment and accomplish tasks. This process is often studied using the protocols of operant conditioning (Thorndike, 1911; Skinner, 1938), in which the animal, free to execute many actions, receives a biologically rewarding outcome if it executes a particular action or sequence of actions. For example, in Edward Thorndike's classic experiments (Thorndike, 1911), a hungry cat was placed in a “puzzle box” and could escape to get food only after it had executed one or several actions, such as pressing a lever and pulling a string. When first placed in the box, the cat would execute many actions, most of which did not affect the box's door, until it happened to press the lever and then pull on the string, after which the box's door opened. With repeated trials, the cat executed fewer of the irrelevant actions, and executed only the actions that led to the door opening.

As with many tasks, Thorndike's puzzle box has massive redundancy in that the outcome can be achieved in many ways (such as by executing irrelevant actions as well as the actions that open the door). Animals resolve this redundancy to a large extent—they are able to achieve the outcome without executing more actions than necessary. We refer to such behavior as the minimal number of actions that achieves an outcome, or, simply, the *minimal action sequence*. Animals are able to discover the minimal action sequence through their own interactions with the environment rather than just from external instruction. How this behavior is learned has been (and is still) the focus of much research in psychology and neuroscience (e.g., Staddon and Cerutti, 2003; Pearce, 2008; Balleine et al., 2009) and, because it describes how learning agents learn from their own experiences, artificial intelligence, and robotics (e.g., Sutton and Barto, 1998; Hart, 2009; Konidaris, 2011).

How are animals able to discover the minimal action sequence? In other words, by what mechanisms do animals discover and reliably execute the minimal action sequence rather than any of the many other action sequences that also achieve the outcome? The achievement of the outcome itself is one obvious signal that can be used to determine if a particular action sequence has achieved the outcome. In addition, in most previous accounts, actions are further evaluated in that actions are associated with a measure of behavior that is higher for actions that lead to achieving the outcome with a smaller total number of actions, and learning mechanisms adjust the tendencies to select actions so as to maximize that measure of behavior. Thus, shorter action sequences that achieve the outcome are preferred because they are determined to be “better” than longer action sequences that achieve the outcome, and the minimal action sequence is the “best” or “optimal” with respect to that measure of behavior. In other words, most previous accounts are concerned not with just the simple evaluation of “was the outcome achieved?”; rather, they are concerned with “how well was the outcome achieved?” where “how well” is in reference to the measure of behavior the learning rule maximizes.

A commonly used computational framework with which to study animal learning processes is a class of optimal control methods called computational reinforcement learning (RL) (Bertsekas and Tsitsiklis, 1996; Sutton and Barto, 1998). RL is inspired in part by animal learning (particularly Thorndike's *Law of Effect*, Chapter 5 of Thorndike, 1911), and neuroscience research in the 1990s (Ljungberg et al., 1992; Schultz et al., 1993) and subsequent research reveal RL's ability to describe biological learning processes (Houk et al., 1995; Schultz et al., 1997) (see also Shah, 2012 or Niv, 2009 for reviews relating RL with psychology and neuroscience). In RL, a learning agent discovers behavioral policies that maximize a measure of behavior that is a function of numerical signals—usually referred to as “reward signals”—delivered by the environment. Typically, a positive numerical signal is delivered when the learning agent achieves the outcome of interest (simulating the biologically rewarding outcome the animal receives when it accomplishes an operant conditioning task), addressing the question “was the outcome achieved?” In addition, the question “how well was the outcome achieved?” is usually addressed by incorporating one or both of the two following types of *cost*. The first type of cost is that every executed action incurs an *explicit action cost* in the form of a negative numerical signal (representing quantities we presume the animal encodes internally that it seeks to minimize, such as muscular effort Pedotti et al., 1978; Fagg et al., 2002; Todorov and Jordan, 2002; Shah et al., 2004). If each executed action incurs a similar explicit cost, the minimal action sequence incurs the fewest negative numerical signals. The second type of cost is that the magnitudes of the numerical signals—in particular, the magnitude of the positive numerical signal delivered when the outcome is achieved—decreases with temporal delay. This *temporal discount* has often been studied in experimental psychology and behavioral economics by presenting an animal with a choice of two actions: one leads to a rewarding outcome after a short delay, the other after a long delay (Samuelson, 1937; Chung, 1965; Logan, 1965; Green and Myerson, 2004). If the two actions lead to an outcome of the same magnitude of reward (such as the same amount of food), the animal is more likely to choose the action associated with the short delay. The temporal delay is thought to add an *implicit cost* by decreasing the perceived magnitude of the reward. If each executed action takes a similar non-zero amount of time to execute, the positive numerical signal upon achieving the outcome is temporally discounted the least with the minimal action sequence.

A learning agent using RL rules modifies its behavior through its own interaction with the environment (executing actions and observing the consequences). *Model-free* or *direct* RL methods use experience exclusively, while other types of RL methods also use models of the environment to behave or modify behavior (Sutton and Barto, 1998; Daw et al., 2005). We focus on model-free methods in this paper. The RL rule generates reinforcement signals based on an error in prediction of the measure of behavior. If an action's consequences result in a higher measure of behavior than expected, reinforcement signals that compare the experienced measure with the expected measure increase the tendency to select that action (the action is *reinforced*), and decrease the tendency if the consequences result in a lower measure than expected. When that measure of behavior is expressed as described in the previous paragraph, an action that leads to achievement of the outcome with a shorter action sequence is considered to be “better” than other actions that also achieve the outcome and the tendency to execute it is greater than the tendency to execute other actions (it is preferred). Thus, RL rules using that measure of behavior focus on “how well was the outcome achieved?”

While mechanisms that use such measures of behavior may account for many types of behaviors, such as acting to maximize rewards received, they may not apply to all types. In particular, Redgrave et al. (Redgrave and Gurney, 2006; Redgrave et al., 2008, 2011, 2013; Stafford et al., 2012; Gurney et al., 2013) discuss how the unexpected occurrence of a salient outcome causes the animal to repeat preceding actions, even if the outcome is not biologically rewarding (see also Horvitz, 2000; Barto et al., 2004). With continued repetition, the animal discovers the actions that achieve the outcome and represents them as a single action in a process referred to as *action discovery*. Importantly, however, the process of action discovery is thought to be driven by the unexpected occurrence of the outcome. As described in detail in Redgrave and Gurney (2006), biological reinforcement signals in action discovery occur too quickly to evaluate behavior beyond an indication of the outcome's occurrence—learning mechanisms in action discovery may be driven by a prediction error regarding the outcome's occurrence, but not an error in prediction of a measure of behavior that is higher for actions that lead to achievement of the outcome with a shorter action sequence. In other words, action discovery is thought to be driven by mechanisms that focus on the simple binary signal of “was the outcome achieved?” and not by the continuous signal of “how well was the outcome achieved?” (Action discovery is considered to be part of broader class of *intrinsically motivated* behavioral development, Barto et al., 2004, 2013b; Redgrave and Gurney, 2006; Oudeyer and Kaplan, 2007; Schmidhuber, 2010; Barto, 2013; Gurney et al., 2013. Also, some neuroscience research suggests that processes that adjust behavioral tendencies and processes that evaluate behavior in terms of measures to be maximized may be mediated by different brain systems, Berridge and Robinson, 1998; Berridge, 2007; Berridge et al., 2009).

If action discovery does not focus on “how well was the outcome achieved?” how can the minimal action sequence be discovered in action discovery? Here, we describe a computational mechanism by which this can occur. We implement *no-cost* learning rules, based on canonical RL rules (Sutton and Barto, 1998), that do not use either of the two types of cost described above. If the outcome is achieved, no-cost learning rules generate reinforcement signals that increase the tendency to execute every action that was executed en route to the outcome, but at a rate that decreases with temporal distance from the outcome. Importantly, in no-cost rules, actions that lead to achievement of the outcome are each associated with the same measure of behavior (indicating that the outcome was achieved) as opposed to a measure of behavior that is higher for actions that lead to achievement of the outcome with a smaller number of actions. No-cost rules focus on the simple evaluation of “was the outcome achieved?” as opposed to “how well was the outcome achieved?” They represent a possible computational mechanism by which the minimal action sequence can be developed that relies on different types of information and processes than previous accounts and is consistent with the process of action discovery.

Recent modeling work (Chersi et al., 2013) has shown that learning rules similar to the no-cost rules we describe in this paper can be used to discover the minimal action sequence. However, they use networks of spiking neurons to investigate neural mechanisms of goal-directed and habitual control and do not directly address the questions we raise in this paper. From the presented results, it is not clear if the learning rule can reliably discover the minimal action sequence if there is massive redundancy, i.e., if a very large number of action sequences of varying lengths can achieve the outcome.

Here, we simulate artificial agents using no-cost learning rules acting within discrete-state discrete-action environments in tasks in which there is massive redundancy and the outcome depends on sequences of more than a few actions. Discrete-state discrete-action environments and tasks are commonly used to describe and evaluate RL algorithms (Sutton and Barto, 1998). The no-cost rules we implement are able to discover and execute, for a temporary yet substantial amount of time, the minimal action sequence. This behavior can be described as optimal with respect to a measure of behavior that is influenced by explicit action costs and/or temporal discounting of numerical signals, but it emerged “for free” without taking these types of cost into account. We go on to describe how such behavior arises from no-cost rules.

Behavior under no-cost rules consists of the minimal action sequence for a substantial period of time; however, if actions continue to be reinforced according to no-cost rules for an extended amount of time, extraneous actions are developed—because any action that leads to achievement of the outcome is associated with the same measure of behavior in no-cost rules, behavior does not converge with extended training. Thus, stable behavior with no-cost rules requires that reinforcement signals be attenuated with a separate process as learning progresses with a separate process. Interestingly, the reinforcement signals, mediated by phasic dopamine neuron activity (Wickens et al., 2003) in biological operant learning, also appear to undergo attenuation (Ljungberg et al., 1992; Schultz et al., 1993, 1997) and a similar process has been proposed in action discovery (Redgrave and Gurney, 2006; Redgrave et al., 2008, 2011, 2013). According to action discovery, reinforcement signals, mediated by phasic dopamine neuron activity, are attenuated by a separate process that is contingent on the ability to predict the occurrence of the outcome. In the work presented here, we do not seek to model the separate prediction process underlying reinforcement attenuation but, rather, we examine resulting behavior if the attenuation were disrupted (e.g., due to disorders in prediction or reinforcement functions). We discuss the significance of this process and no-cost learning rules in relation to other theories of reinforcement attenuation, namely, descriptions of phasic dopamine neuron activity in terms of an error in prediction of a measure of behavior that is different for different actions (Houk et al., 1995; Schultz et al., 1997), in the Discussion section. Elements of this work have been presented in poster format (Shah and Gurney, 2011).

## 2. Methods

### 2.1. Environment

**Figure 1:**
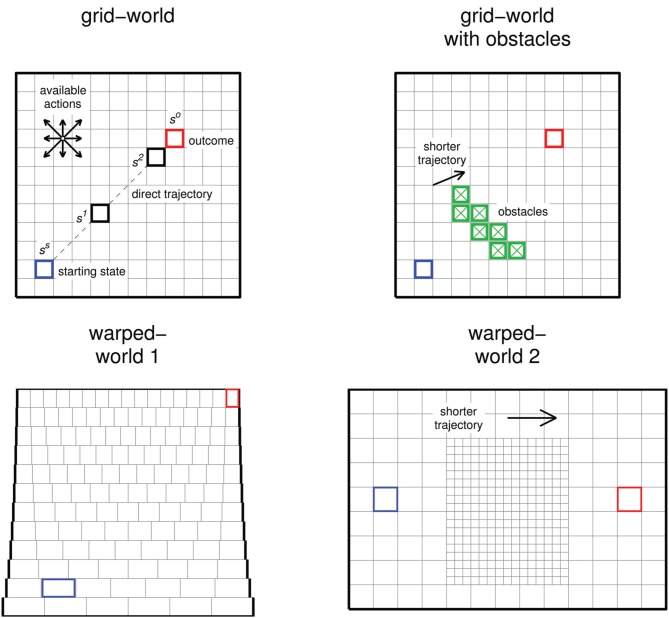
**Illustrations of the environments we use**. Each “state” is represented as a small square or rectangle within the environment. Actions can be executed that transition the agent from one state to another (see text). In all environments, the state colored blue (labeled *s^s^*) is the starting state and the state colored red (*s^o^*) is the goal state and represents achievement of the outcome. In the grid-world (top left), states labeled *s*^1^ and *s*^2^ are referred to in the text. In the grid-world with obstacles, transitions into obstacle states (highlighted in green and with a “×”) are prevented. In both grid-worlds, four cardinal and four diagonal actions are available from each state; actions are deterministic. In warped-world 1 (bottom left), only four cardinal actions are available. Because states are not aligned vertically, actions to the north or south stochastically transport the agent into one of the two states that overlap the current state, weighted by the relative amounts the states overlap the current state (see Supplementary Material for details). In warped-world 2, four cardinal and four diagonal actions are available, but actions between “big” and “small” states are different than actions between big and big states and actions between small and small states (see Supplementary Material for details).

We subject learning agents to tasks in discrete-state discrete-action environments (Figure 1) in which a “state” is an abstract representation of the current situation or context from which to take an action, and an “action” causes a transition from one state to another. The environment is Markov: the effect of an action depends only on the current state and not on previous states visited. Such environments can be represented in different ways. A typical representation used to demonstrate and evaluate RL algorithms is the *grid-world* environment (Figure 1 top row) (Sutton and Barto, 1998), in which states are visually represented in a spatial grid, states that can be reached from each other with one action are placed next to each other, and the effects of an action are analogous to movements in the grid-world. Thus, states that can be reached from each other with a small number of actions are placed closer together than states that require a larger number of actions to be reached from each other. By using such a representation, a behavioral trajectory that follows “the minimal action sequence” from one state to another is readily apparent as the shortest trajectory when visually illustrated. Although the grid-world representation suggests a maze to test navigational abilities, it is misleading to think of it in this way. It merely provides a visually accessible representation of an abstract sequential decision task. Using Thorndike's puzzle box as an example, suppose that the cat scratched itself, pulled the chain, pawed at the door, batted the wall, licked its paw, and then pressed the lever to open the door. This sequence of actions would be represented as a longer trajectory than if the cat only pulled the chain and pressed the lever to open the door.

In addition, the tasks we simulate are *episodic* (Sutton and Barto, 1998) in that experiences are clearly segregated into subsequences referred to as “episodes” or “trials,” analogous to trials in most types of operant conditioning experiments (Thorndike, 1911; Skinner, 1938). In simulations, the end of one trial is followed by the beginning of the next trial, time steps refer to the time step within a trial, and experiences generated during a trial influence learning during the current trial but not previous trials. Also, in our simulations, trials are not of a fixed length. Rather, as explained below, a trial terminates when a particular goal state is reached or when a maximum number of time steps has elapsed (a “time-out”). As discussed in Sutton and Barto (1987), this explicit segregation is a simplification as animal learning processes may not incorporate mechanisms that are dependent on the concept of an episode or trial. An effect similar to an explicit segregation can be accomplished by providing an explicit indication at the start of a trial (as is often the case in experimental tasks) or by imposing a lengthy delay between trials (as, for example, Izhikevich, 2007 does in simulation), but the explicit segregation of the episodic task framework is mathematically simpler (Sutton and Barto, 1998) for our purposes. Episodic tasks are commonly used in RL models of human and animal tasks (e.g., Daw et al., 2005; Shah and Barto, 2009; Gläscher et al., 2010; Knox and Stone, 2012) and to assess artificial learning algorithms in general (e.g., Sutton and Barto, 1998; Konidaris and Barto, 2009).

In the basic *grid-world* task (Figure 1 top left), states are arranged in a 12 × 12 grid. At the beginning of each trial, the current time step, *t*, is set to 1 and the agent is placed in a fixed starting state, *s^s^*, highlighted in blue in Figure 1. At each time step, the agent chooses an action, *a*, from the set of possible actions: four cardinal and four diagonal actions in the grid-world. Action effects are deterministic, and actions that would cause a transition off the grid result in no change in state. The trial terminates if the agent transitions into state *s^o^* (highlighted in red), which signifies the achievement of the outcome, or after 115 time steps in the grid-world environments. This somewhat arbitrary “time-out,” about 10% × number of states × number of possible actions, allows for massive redundancy in that there is a very large number of action sequences of varying lengths that can reach *s^o^* from *s^s^* within the time-out. (In contrast, if the time-out were the same as the minimal number of actions that could achieve the outcome, the task would be non-redundant: only behavior that uses the minimal number of actions to achieve the outcome could achieve the outcome within the time-out). *T* refers to the last time step of a trial and is always ≥ the minimal number of actions it takes to achieve the outcome and ≤ the time-out. In addition, a numerical signal, *r_t_*, is delivered at each time step (this signal is different in with-cost vs. no-cost measures of behavior, as described below).

The *grid-world with obstacles* (Figure 1 top right) is the same as the grid-world except that transition into obstacle states (green squares with a “×”) is prevented. The obstacles prevent a spatially direct trajectory from *s^s^* to *s^o^*. In the grid-world without obstacles, short-length trajectories are easily reached from each other, so the chances of getting stuck in a local minimum are not high. We use the grid-world with obstacles to examine behavior in environments in which some short-length trajectories (i.e., above and below the obstacles) are not easily reached from each other, in which case the chances of getting stuck in a local minimum have increased.

While these tasks are not strictly navigation tasks, they can serve as abstract representations of tasks with some underlying geometric structure. To examine how behavior developed under different learning rules may be interpreted in such cases, we examine behavior in two spatially “warped” environments as well. In *warped-world 1* (Figure 1 bottom left), the number of states along the horizontal dimension is larger at higher vertical locations than that at lower vertical locations. Thus, states do not represent the underlying spatial geometry uniformly, e.g., states at higher vertical locations represent a smaller spatial area than states at lower vertical locations. Also, only the four cardinal actions are available. Because the states are not aligned vertically, the effects of actions north and south in warped-world 1 are stochastic (see Figure caption and Supplementary Material for details). In *warped-world 2* (Figure 1 bottom right), “small” states, which are in the middle and lower areas of the environment, represent smaller spatial areas than do “big” states, which are in the outer areas of the environment. Cardinal and diagonal actions are available; actions between small and big states have slightly different effects than do actions from big to big states or actions from small to small states (see Supplementary Material for details). The time-outs for warped-worlds 1 and 2 are 276 and 286 steps, respectively.

We use the warped-worlds to examine possible ways by which spatially indirect behavior can be accounted if overall behavior was observed but the underlying representations of states and actions were not known. One possible account of spatially indirect behavior is that the state representation is spatially uniform (as in grid-worlds), the animal assigns a higher cost to particular actions made a particular locations, and behavior is developed with a learning rule that incorporates explicit action costs. For example, a behavioral trajectory that travels east and then north may be taken as evidence that trajectories that travel north and then east are more costly, or that horizontal actions executed at higher vertical locations are more costly than horizontal actions executed at lower vertical locations. Similarly, trajectories that avoid the center of an environment may be taken as evidence that trajectories that go through the middle of the environment incur greater action costs.

We suggest that spatially indirect behavior can also be accounted for with mechanisms that do not incorporate explicit action costs. Instead, such behavior may emerge from nonuniform representations of the environment already in place through prior experience and developmental processes. Spatial representation and cognition is a topic of much current research (Moser et al., 2008; Chen et al., 2014; Willis et al., 2014) and nonuniform representations occur in many central nervous system structures involved with different modalities (van Essen et al., 1984; Curcio et al., 1990; Kurtzer et al., 2006; Graziano and Aflalo, 2007; Scott, 2008; Lillicrap and Scott, 2013). We use the warped-worlds to examine behavior resulting from mechanisms that do not incorporate explicit action costs if the state representation is nonuniform in the spatial domain.

### 2.2. *With-cost* vs. *no-cost* measures of behavior

A measure of behavior, often referred to as the *return* in RL (Sutton and Barto, 1998), is determined by *r_t_*, the numerical signal delivered at each time step. The return at time *t* of a trial is the sum of these signals from *t* + 1 to the end of trial: $R_{t}=\sum_{i\;=\;t\;+\;1}^{T}r_{i}$, where *T* indicates the last time step of a trial. (Recall that *t* = 1 at the beginning of a trial and, because a trial terminates when *s^o^* is achieved, *T* depends on the number of actions taken during the trial, and *T* will always be less than or equal to the time-out). We differentiate *with-cost* and *no-cost* measures of behavior by the information communicated by *r_t_*.

#### 2.2.1. With-cost measures

Under *with-cost* measures of behavior, *r_t_* is used to associate different actions with different measures of behavior if they lead to achievement of the outcome with different numbers of actions. *r_t_* is an explicit action-dependent negative numerical signal (a “cost”) of −1 if a cardinal action was selected at time *t* and −$\sqrt{2}$ if a diagonal action was selected (e.g., Sutton and Barto, 1998; Shah and Barto, 2009). Transition into *s^o^* delivers a positive numerical signal of *r_o_* = +20 instead of the action-dependent cost. Because *R_t_* is the sum of action-dependent costs (and *r_o_* if *s^o^* is achieved), *R_t_* can take on a range of values: *R_t_* is different for each *t* and is lower for *t* earlier in the trial. In the grid-world, the action sequence from *s^s^* to *s^o^* that is associated with the highest return follows the spatially direct trajectory (dashed line in Figure 1), which is also the minimal action sequence. Longer action sequences are associated with a lower return because more negative signals contribute to the return. In this way, the with-cost measure of behavior is often different for different actions selected from a particular state or different action sequences and can be used in learning rules that focus on “how well was the outcome achieved?”

We note that the return can also be expressed as $R_{t}=\sum_{i\;=\;t\;+\;1}^{T}\gamma^{i-t-1}r_{i}$ (Sutton and Barto, 1998), where γ captures the effect of temporal discounting: if 0 ≤ γ < 1, then numerical signals delivered with a long delay (*t* + delay) will have less weight on *R_t_* than signals delivered with a short delay. For example, if *r_t_* = *r_o_* = +20 if the outcome is achieved but *r_t_* = 0 otherwise, and γ < 1, then the return will be less if the outcome is achieved after a larger number of actions than if the outcome is achieved after a smaller number of actions. As described in the Introduction, temporal discounting can also be thought of as a type of cost. Because explicit action costs and temporal discounting have similar effects on the return—longer action sequences that achieve the outcome are associated with a lower measure of behavior than shorter action sequences that achieve the outcome—we include only explicit action costs in this paper for simplicity, i.e., if γ were included in the equations, γ would be set to 1.

#### 2.2.2. No-cost measures

Under *no-cost* measures of behavior, *r_t_* = 0 at every time step except if *s^o^* is reached, at which point *r_t_* = *r_o_* = +20 (and, as above, there is no temporal discounting of *r_t_*). Thus, under no-cost measures of behavior, *R_t_* can take on only two values (0 or *r_o_* = +20) and is the same for every *t* during the trial: 0 if *s^o^* was not achieved during the trial, and *r_o_* = +20 if *s^o^* was achieved during the trial. In this way, the no-cost measure of behavior is the same for any action sequence that achieves the outcome and can be used in learning rules that focus on “was the outcome achieved?”

### 2.3. Action selection

The tendency to select action *a* when in state *s* is represented by *Q*(*s*, *a*). Actions are selected stochastically according to their relative tendencies (via a “softmax” function, as described in Sutton and Barto, 1998):

(1) $$p(s,a)=\frac{e^{Q(s,a)/\tau }}{\sum_{i=1}^{A}e^{Q(s,a_{i})/\tau }},$$

where *p*(*s*, *a*) is the probability of selecting action *a* from state *s* and τ (= 1.5) controls the stochasticity. Initial *Q*(*s*, *a*) are set to zero. Learning rules described below modify *Q*(*s*, *a*) for each visited state and action based on experience. If the tendency to select an action is increased (if *Q*(*s*, *a*) increases), that action is said to be *reinforced*.

### 2.4. Monte Carlo and temporal difference learning rules

We implement several learning rules expressed in the form of one of two types of RL rules (Sutton and Barto, 1998) that modify *Q*(*s*, *a*) based on experience. The first type, called Monte Carlo (MC) rules, use *R_t_* directly to deliver reinforcement signals only at the end of the trial. The state visited at time *t* is denoted *s_t_* and the action executed from that state is denoted *a_t_*. *Q*(*s_t_*, *a_t_*) for each visited (*s_t_*, *a_t_*) is modified at the end of a trial with the actual *R_t_* experienced during the trial:

(2) $$Q(s_{t},a_{t})\leftarrow Q(s_{t},a_{t})+\alpha \lambda^{T-t-1}\left[R_{t}-Q(s_{t},a_{t})\right],$$

where α (= 0.1) is a step-size and λ (0 ≤ λ ≤ 1) defines an eligibility trace (Pavlov, 1927; Sutton and Barto, 1981, 1998; Klopf, 1982; Wörgötter and Porr, 2005). The eligibility trace allows for *Q*(*s_t_*, *a_t_*) for *t* before *T* (the last time step of the trial) to be modified and controls the rate at which it is modified. If 0 < λ < 1, the eligibility trace is decaying in time and *Q*(*s_t_*, *a_t_*) for each (*s_t_*, *a_t_*) is modified with a rate that is lower for *t* far from *T*, i.e., *Q*(*s_t_*, *a_t_*) for *t* early in a trial is modified at a lower rate than *Q*(*s_t_*, *a_t_*) for *t* later in a trial. If λ = 1, the eligibility trace is non-decaying and *Q*(*s_t_*, *a_t_*) for each (*s_t_*, *a_t_*) is modified at the same rate. We refer to these rules as MC(λ).

The second type of learning rule is a temporal difference (TD) rule (Sutton, 1988; Sutton and Barto, 1998), in which *Q*(*s*_*t* − 1_, *a*_*t* − 1_) is modified at every time step with *r_t_* and *Q*(*s_t_*, *a_t_*):

(3) $$\begin{array}{l} Q(s_{t-1},a_{t-1})\leftarrow Q(s_{t-1},a_{t-1})+\alpha [r_{t}+Q(s_{t},a_{t})\\ \;-\;Q(s_{t-1},a_{t-1})] \end{array}$$

(this particular formulation is the “SARSA” learning rule, Rummery and Niranjan, 1994). Each *Q*(*s*_*t* − 1_, *a*_*t* − 1_) is modified to be closer to *r_t_* + *Q*(*s_t_*, *a_t_*) (and thus indirectly to *R_t_*). (Note that there is no temporal discount term). This rule does not have an eligibility trace. However, it can be considered a special case of similar rules that do have eligibility traces (Sutton and Barto, 1998), but with λ = 0. We thus refer to it as TD(0).

### 2.5 With-cost vs. no-cost learning rules

*Q*(*s*, *a*) for each (*s*, *a*) visited during a trial are modified by learning rules toward the measure of behavior to be expected if action *a* were execute from state *s*.

#### 2.5.1. With-cost learning rules

For comparison purposes, we implement two standard RL algorithms (Sutton and Barto, 1998) using the with-cost measure of behavior: MC(1) (where λ = 1) and TD(0), referred to here as *with-cost rules*.

When modified according to with-cost rules, *Q*(*s_t_*, *a_t_*) for each *Q*(*s_t_*, *a_t_*) visited during a trial is modified toward the return (*R_t_*) according to the with-cost measure of behavior. The with-cost measure of behavior includes the sum of explicit action costs received during the trial after selecting action *a_t_* from state *s_t_* and, if the outcome is achieved during the trial, *r_o_* = +20. (*Q*(*s*, *a*) is modified toward the actual experienced return in MC rules, and the next *r_t_* and *Q*(*s*, *a*) in TD rules). *R_t_* can thus take on a range of values under with-cost measures of behavior and *Q*(*s*, *a*) will converge to different values for different actions: *Q*(*s*, *a*) for the action that leads to achievement of the outcome with a smaller number of actions will be higher than *Q*(*s*, *a*) for the action that leads to achievement of the outcome with a larger number of actions. (As described in the Introduction and earlier in the Methods, temporally discounting *r_t_* would also have this effect). Because they use with-cost measures of behavior, with-cost learning rules focus on “how well was the outcome achieved?”

#### 2.5.2. No-cost learning rules

We suggest that a simple rule using a no-cost measure of behavior (which is not influenced by explicit action costs) would be an MC rule because the reinforcement signal is generated at only one time step (*T*) and only one return need be generated. We also suggest that it use a decaying eligibility trace. We thus implement an MC rule with a no-cost measure and λ = 0.7, referred to as ncMC(0.7) (where “nc” indicates “no-cost”). We implement two other *no-cost rules* for comparison purposes: ncMC(1) (with λ = 1) and ncTD(0).

When modified according to no-cost rules, *Q*(*s*, *a*) is not modified toward a return that incorporates explicit action costs. Instead, the return can take on one of only two values under no-cost measures (0 if *s^o^* is not achieved or *r_o_* = +20 if *s^o^* is achieved), and each *Q*(*s*, *a*) during a trial is modified toward the same value. No-cost rules use no-cost measures of behavior and focus on “was the outcome achieved?”

Because *Q*(*s*, *a*) for each state and action approach the same value under no-cost rules in our simulations, and actions are selected stochastically according to *Q*(*s*, *a*), behavior according to *Q*(*s*, *a*) modified with no-cost rules does not converge with extended experience. Rather, *Q*(*s*, *a*) will approach *r_o_* for all states and actions in our simulations and, eventually, each action will be chosen with equal probability. In order for behavior under no-cost rules to stabilize, another process must attenuate reinforcement signals. We do not model this process here so as to describe the behavioral patterns that would result from inappropriate continued reinforcement. (Also, note that in our simulations, there is no state or action from which it is not possible to achieve *s^o^*. In tasks and environments in which there do exist states and actions from which it is not possible to achieve *s^o^*, *Q*(*s*, *a*) for those states and actions will remain at 0).

### 2.6. Experiments

A *run* consists of an agent undergoing 200,000 trials. (A large number of trials was chosen so as to better expose the effects of reinforcement for an extended period of time under the different rules). Twenty runs for each learning rule were conducted using the grid-world environment. Behavior was examined at *test points* (every 100 trials for the first 1000 trials; every 1000 trials after that), during which α = 0 and five *sample trials* using the *Q*-values from the test point were run. Thus, 100 sample trials (five sample trials for each of the twenty runs) for each test point were used to report behavior.

In addition, twenty runs using ncMC(0.7) were conducted for the grid-world with obstacles and the two warped-worlds.

## 3. Results

### 3.1. Discovery of the minimal action sequence in the grid-world

**Figure 2:**
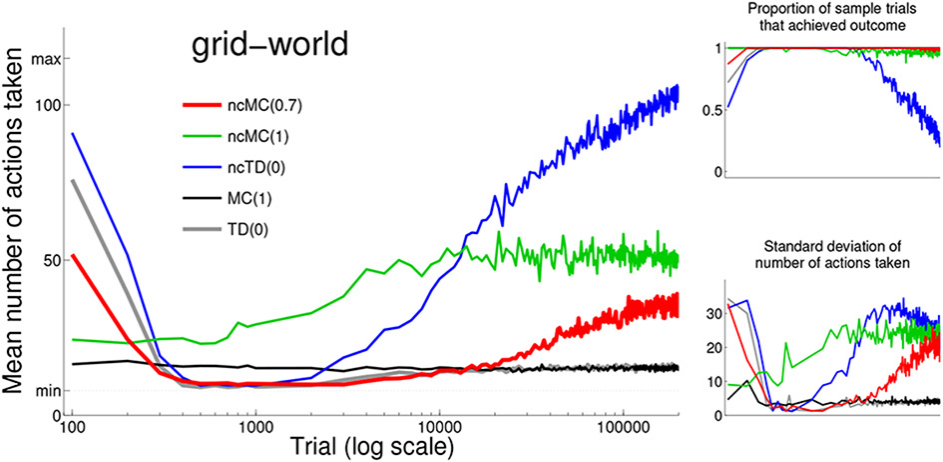
**Left:** mean (across all sample trials) number of actions at each test point for each learning rule (see legend for color scheme). Trial time-out is indicated by “max” (115) on the vertical axis. The minimum number of actions needed to achieve *s^o^* (8) is indicated by “min.” Note that the horizontal axis uses a log scale. **Bottom right:** standard deviation of the number of actions at each test point for each learning rule. **Top right:** proportion of sample trials that achieved *s^o^*.

We use the word “behavior” to refer to the agent traversing a series of states by executing a sequence of actions. For the grid-world (Figure 1 top left), behavior that follows the spatially direct trajectory (dashed line) is the minimal action sequence because it achieves the outcome—reaches *s^o^* from *s^s^*—using the minimal number of actions. If an outsider observed this behavior, and was not aware of the mechanisms by which it was developed, he may describe it as optimal with respect to a measure of behavior that is higher for actions that lead to achieving the outcome with a smaller total number of actions, such as a measure of behavior in which every executed action is accompanied by an explicit cost (negative numerical signal) (as described in the Methods). Figure 2 left shows the mean (across all sample trials) number of actions at each test point for each learning rule. Note that standard deviation (Figure 2 bottom right) is very low at sample trials for which the mean number of actions is very low. Figure 2 top right shows the proportion of sample trials that achieved the outcome at each test point for each learning rule.

As seen in Figure 2 and consistent with descriptions in Sutton and Barto (1998), the two standard with-cost rules, MC(1) and TD(0), develop the minimal action sequence in that behavior converges to a low number actions (stochasticity inherent in action selection prevents any rule from executing only the minimal action sequence). This is unsurprising because they incorporate the with-cost measure of behavior in which each action incurs an explicit cost.

Behavior under no-cost rule ncMC(1) reliably achieves *s^o^*, but ncMC(1) was not able to discover and execute (on average) behavior that uses a low number of actions. No-cost rule ncMC(0.7) was able to discover and execute, for trials 400 to 11,000, the minimal action sequence. Similarly, no-cost rule ncTD(0) was able to discover and execute, for trials 400–2000, the minimal action sequence. Although the minimal action sequence can be described as optimal with respect to the with-cost measure of behavior in that it reliably executes actions associated with the highest with-cost measure of behavior (the minimal action sequence), it is important to note that ncMC(0.7) and ncTD(0) use the no-cost measure of behavior, in which any action sequence that achieves the outcome is associated with the same measure of behavior. We explain how no-cost rules discover the minimal action sequence in the next subsection.

Behavior under with-cost rules converges with continued reinforcement for an extensive period of time. Behavior under no-cost rules does not; rather, the mean number of actions increases with continued reinforcement for an extensive period of time (up to 200,000 trials in our simulations).

### 3.2. How no-cost rules discover the minimal action sequence

The ability of ncMC(0.7) to discover the minimal action sequence can be understood by examining how the decaying eligibility trace (λ < 1, see Methods) affects the rate at which *Q*(*s*, *a*) for each action executed at each state visited en route to the outcome is modified. Let *s_t_* be the state visited at time *t*, and *a_t_* be the action executed from state *s_t_*. Recall that, under ncMC(0.7), *Q*(*s_t_*, *a_t_*) for each visited (*s_t_*, *a_t_*) is modified toward the same value at each trial: *r_o_* = +20 if *s^o^* was achieved, 0 if not. (In contrast, in with-cost rules, which use the with-cost measure of behavior, *Q*(*s_t_*, *a_t_*) for each visited (*s_t_*, *a_t_*) is modified toward different values because they lead to action sequences of different lengths.) However, because λ < 1, the rate at which *Q*(*s_t_*, *a_t_*) is modified by ncMC(0.7) depends on the temporal distance of *t* from *T* (where *T* indicates the time step at the end of the trial): *Q*(*s_t_*, *a_t_*) for *t* early in a trial (and thus far from *T*) are modified at a lower rate than *Q*(*s_t_*, *a_t_*) for *t* late in a trial. This has the effect of reinforcing actions that lead to shorter action sequences that achieve the outcome at a greater rate than actions that lead to longer action sequences that achieve the outcome, even though all *Q*(*s_t_*, *a_t_*) are modified toward the same value.

**Figure 3:**
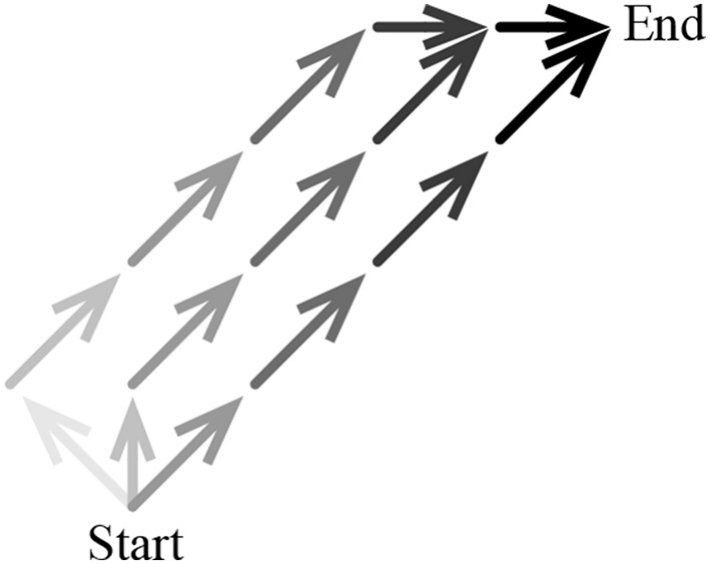
**Simplified schematic of three sequences of actions from “Start” to “End.”** Each action is represented by an arrow; the darker the arrow, the greater the rate at which the tendency to select the action is modified.

This idea is illustrated in Figure 3, which is a simplified schematic of three sequences of actions from one state (“Start”) to another (“End”). The darker the arrow representing the action, the greater the rate at which that action is reinforced if the outcome is achieved: actions executed at a closer temporal distance to End are reinforced at a greater rate than actions executed at a further temporal distance to End. As in the grid-world, the minimal action sequence consists of taking the action northeast to move directly from Start to End (right-most action sequence in Figure 3). A slightly longer action sequence involves taking action north from Start and then moving directly to End (middle action sequence). The longest of the three action sequences involves taking action northwest from Start and then moving directly to End (left-most action sequence). Because taking action north from Start leads to a longer action sequence than taking northeast from Start, action north from Start is reinforced at a lower rate than action northeast from Start. Similarly, action northwest from Start is reinforced at an even lower rate.

Under ncMC(0.7), all actions that were executed during trials in which the outcome was achieved are reinforced toward the same measure of behavior (*r_o_* = +20), but those that lead to shorter action sequences are reinforced at a greater rate than those that lead to longer action sequences. In other words, the minimal action sequence is reinforced at a greater rate than all other sequences that achieve the outcome. Also, because action selection—behavior—is based on a softmax function of *Q*(*s*, *a*) (see Methods), actions associated with a higher *Q* (the minimal action sequence) are more likely to be executed for a period of time. Thus, ncMC(0.7) discovers and executes, for the vast majority of the first 11,000 trials, the minimal action sequence. (As described later, because each *Q*(*s_t_*, *a_t_*) is modified toward *r_o_* = +20 if the outcome is achieved, eventually all action sequences will be equally likely to be executed—extraneous actions will be selected with continued reinforcement and extensive experience).

Similar reasoning explains how ncTD(0) discovers the minimal action sequence: because information at time *t* (*r_t_* and *Q*(*s_t_*, *a_t_*) for the TD rules in this paper) are used to modify *Q*(*s*_*t* − 1_, *a*_*t* − 1_) in the TD rules we use (Sutton, 1988; Rummery and Niranjan, 1994; Sutton and Barto, 1998), information available at *t* late in a trial must propagate over several trials to (*s_t_*, *a_t_*) visited at *t* earlier in a trial. Thus, *Q*(*s_t_*, *a_t_*) for *t* later in a trial are modified at a greater rate than that for *t* earlier in a trial under ncTD(0) as well. (This feature also offers an explanation for the observation that behavior as developed by with-cost rule TD(0) actually uses fewer actions (on average) from trials 400 to 2000 than at later trials, Figure 2).

As demonstrated with behavior developed under rule ncMC(1), simply reinforcing behavior that achieves *s^o^*, and decreasing the tendency to select behavior that does not achieve *s^o^* within the time-out, provides a small bias toward—but not reaching—the minimal action sequence. ncMC(0.7) and ncTD(0) reinforce all actions that achieve *s^o^* as well, but, because the rate of reinforcement is greater for actions executed in closer temporal proximity to *T*, ncMC(0.7) and ncTD(0) can discover and execute the minimal action sequence for a temporary but substantial period of time.

**Figure 4:**
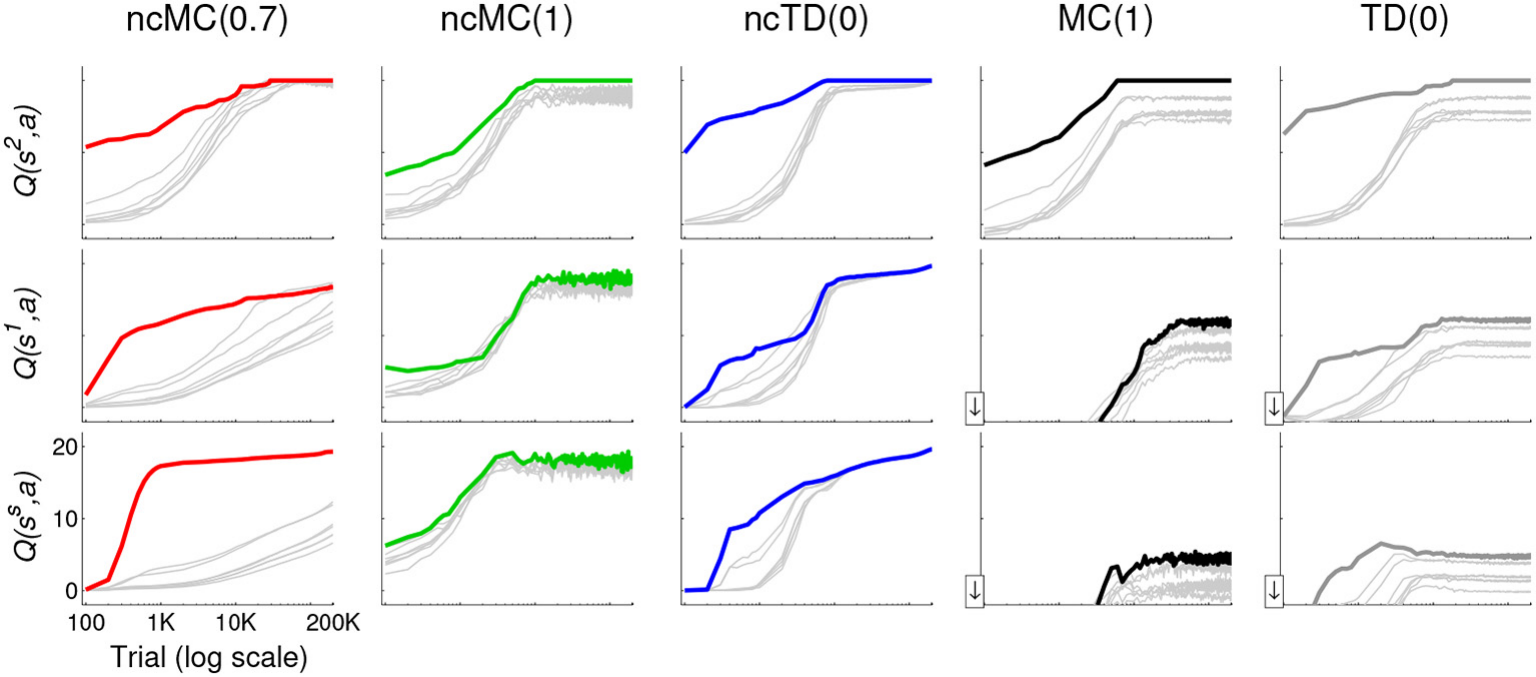
**Mean (across the 20 runs) *Q*(*s*, *a*) at each test point for states *s^s^*, *s*^1^, and *s*^2^ (highlighted in Figure 1) for learning agents in the grid-world using the different learning rules**. The learning rules are indicated at the top. *Q*(*s*, *a*) for *a* = northeast, which is the action that leads to the shortest action sequence in each case, is drawn with a thick line in color according to the legend in Figure 1. That of all other actions are drawn with thin gray lines. The horizontal axis (log scale) is the same in each graph, as is the vertical axis. The downward arrow at the bottom of the vertical axis in the graphs in the lower right indicates that *Q*(*s*, *a*) in these graphs actually fall below the lower limit of the vertical axis (i.e., they are negative) during early trials. However, we cut off these graphs to enable a visually clearer comparison of *Q*(*s*, *a*) evolution in the latter stages of training under the different learning rules.

These concepts are also illustrated in Figure 4, which graphs, for each learning rule, the mean *Q*(*s*, *a*) for each action at states *s^s^*, *s*^1^, and *s*^2^ (highlighted in Figure 1 top left) as a function of trial number. State *s*^2^ is spatially close to *s^o^* (the outcome); *s^s^* (the starting state) is spatially far from *s^o^*; and *s*^1^ is in between. *Q*(*s*, *a*) for the most direct action (northeast for each of the three states) is highlighted in color (according to the legend in Figure 2). Actions executed from states spatially closer to *s^o^* are more likely to be executed at *t* closer to *T* than those from states farther from *s^o^*. Thus, if the outcome is achieved, actions from *s*^2^ are reinforced at a greater rate than those from *s*^1^, which are reinforced at a greater rate than those from *s^s^* (Figure 4). Also, in all no-cost rules, at states *s^s^*, *s*^1^, and *s*^2^, action northeast is reinforced at a greater rate than other actions (this effect is much stronger for rules ncTD(0.7) and ncTD(0) than for ncMC(1)).

The use of stochastic action selection allows all (*s*, *a*) to eventually be visited many times. As a result, *Q*(*s*, *a*) for each (*s*, *a*) gets modified toward *r_o_* = +20 if *s^o^* was achieved (0 if not) under no-cost rules. Thus, eventually all *Q*(*s*, *a*) will be close to +20 with continued reinforcement (trials during which *s^o^* is not achieved prevent them from reaching +20). Because actions are selected stochastically based on *Q*(*s*, *a*), with extensive experience and continued reinforcement, all actions will eventually be equally likely to be selected and behavior as developed by no-cost rules will deviate from the minimal action sequence. This can be seen in Figure 4, left three columns. In contrast, because *Q*(*s*, *a*) as developed by with-cost rules converge to different values, depending on the number of actions executed subsequently, behavior as developed by with-cost rules stabilizes to close to the minimal action sequence even with continued reinforcement (Figure 4 right two columns).

### 3.3. Pattern of development of extraneous actions

**Figure 5:**
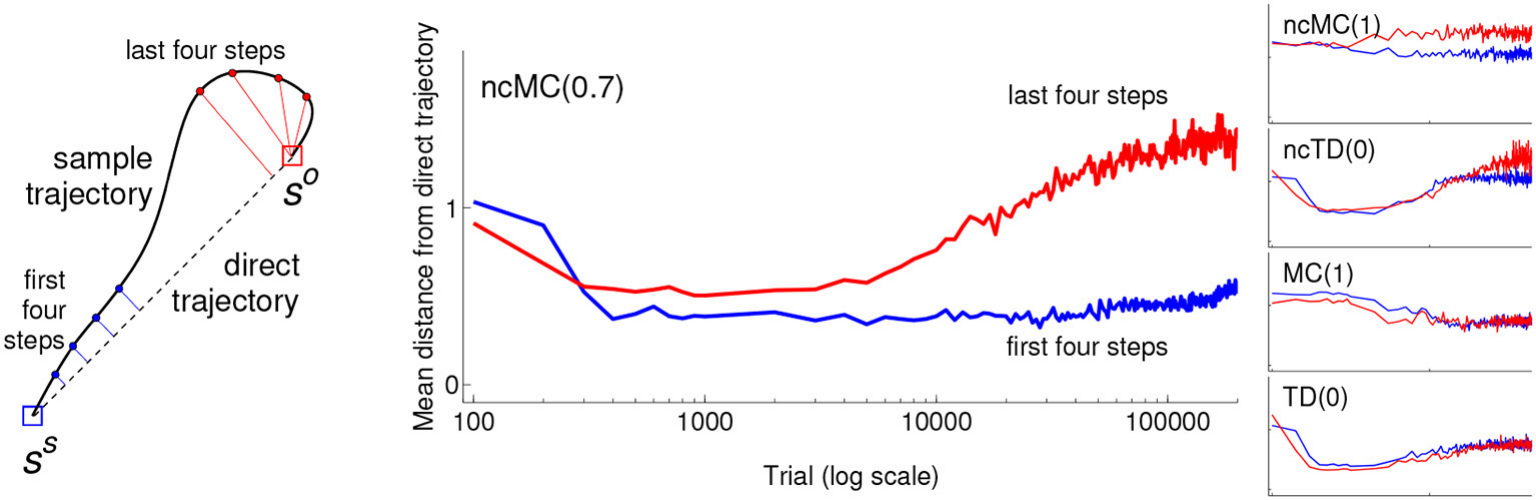
**Mean (across sample trials that achieved *s^o^*) shortest distance from the line segment between *s^s^* and *s^o^* of the first four steps after the start of the trial (blue) and the last four steps before *s^o^* was achieved (red) for learning agents in the grid-world using the different learning rules**. Sample trials that did not achieve *s^o^* were excluded (otherwise the distance measure would necessarily be shorter for the first four steps because the agents start every trial at *s^s^*, but they are not restricted to end every trial at *s^o^*). **Left:** schematic illustrating the distance measures. This schematic uses a continuous trajectory to more-clearly illustrate that the distance measures are based on the first four steps and last four steps of a trajectory; actual trajectories are a series of straight line segments. **Center:** The mean distance for agents using rule ncMC(0.7) (note the horizontal axis uses a log scale). **Right:** That for agents using the other rules. Each graph uses the same horizontal and vertical scales and limits.

**Figure 6:**
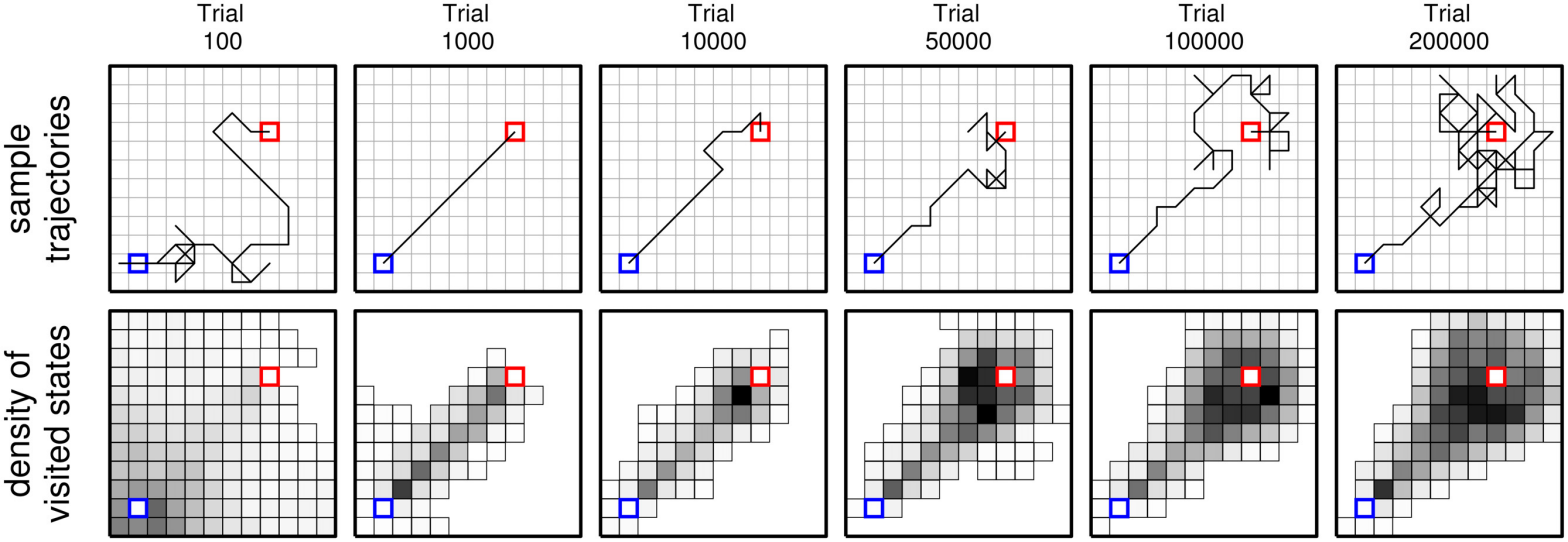
**Sample trajectories (top row) and density of visited states (bottom) (across all sample trials) for learning agents in the grid-world using learning rule ncMC(0.7) at different test points (indicated at the top)**. For indicating density of visited states, each state was colored in gray scale: the darker the color, the larger the number of times that state was visited across all sample trials. States *s^s^* (blue) and *s^o^* (red) were not colored in, and states that were not visited were not drawn.

Under ncMC(0.7), *Q*(*s*, *a*) increases toward *r_o_* = +20 (if *s^o^* is reached) at a greater rate for (*s*, *a*) visited closer *T* (the last time step of a trial) than for (*s*, *a*) visited further from *T* (Figures 3, 4). Thus, if reinforcement under ncMC(0.7) continues for an extended amount of time, extraneous actions will be selected at states closer to *s^o^* (which is a termination condition for a trial) earlier in experience than at states closer to *s^s^*. Figures 5, 6 illustrate this pattern.

Figure 5 shows, for sample trials that achieved *s^o^* in the grid-world, the mean shortest distance from the line segment between *s^s^* and *s^o^* of the first four visited states (after *s^s^*) of the trial and that of the last four states (before *s^o^*) of the trial. Behavior developed by ncMC(0.7) (large center panel) displays a clear pattern in which the mean distance for the last four increases at a greater rate than that of the first four. Behavior generated under ncTD(0) (second from top on the right) shows a similar, but weaker, pattern. Such a pattern is not clearly apparent in behavior generated under the other rules. Other ways of seeing this pattern are illustrated in Figure 6, which shows sample trajectories (top row) and density of visited states (bottom) for sample trials that achieved *s^o^* under ncMC(0.7) at different test points.

The distance metrics in Figure 5 for with-cost rule TD(0) (bottom right) also illustrates the observation made earlier that the bias toward the minimal action sequence for behavior generated under this rule is stronger at early trials (400–2000) than at later trials, even though the rule converges to executing short action sequences. In addition, the metrics reveal a slightly greater deviation from the direct trajectory for the last four steps of behavior generated under rule ncMC(1) than that for the first four steps. This suggests that additional factors may also influence these metrics. For example, the fact that states visited at late *t* depend on actions selected at earlier *t* implies that, due to error accumulation, the agent is simply more likely to visit states away from the direct trajectory at later *t* than at earlier *t*. (Recall that this analysis is confined to sample trials in which *s^o^* was achieved, so the effects of sample trials in which *s^o^* was not achieved are not included). However, this factor does not account for the clear pattern exhibited by behavior under rule ncMC(0.7) and the somewhat weaker but similar pattern exhibited by behavior under rule ncTD(0).

### 3.4. Behavior under ncMC(0.7) in different environments

**Figure 7:**
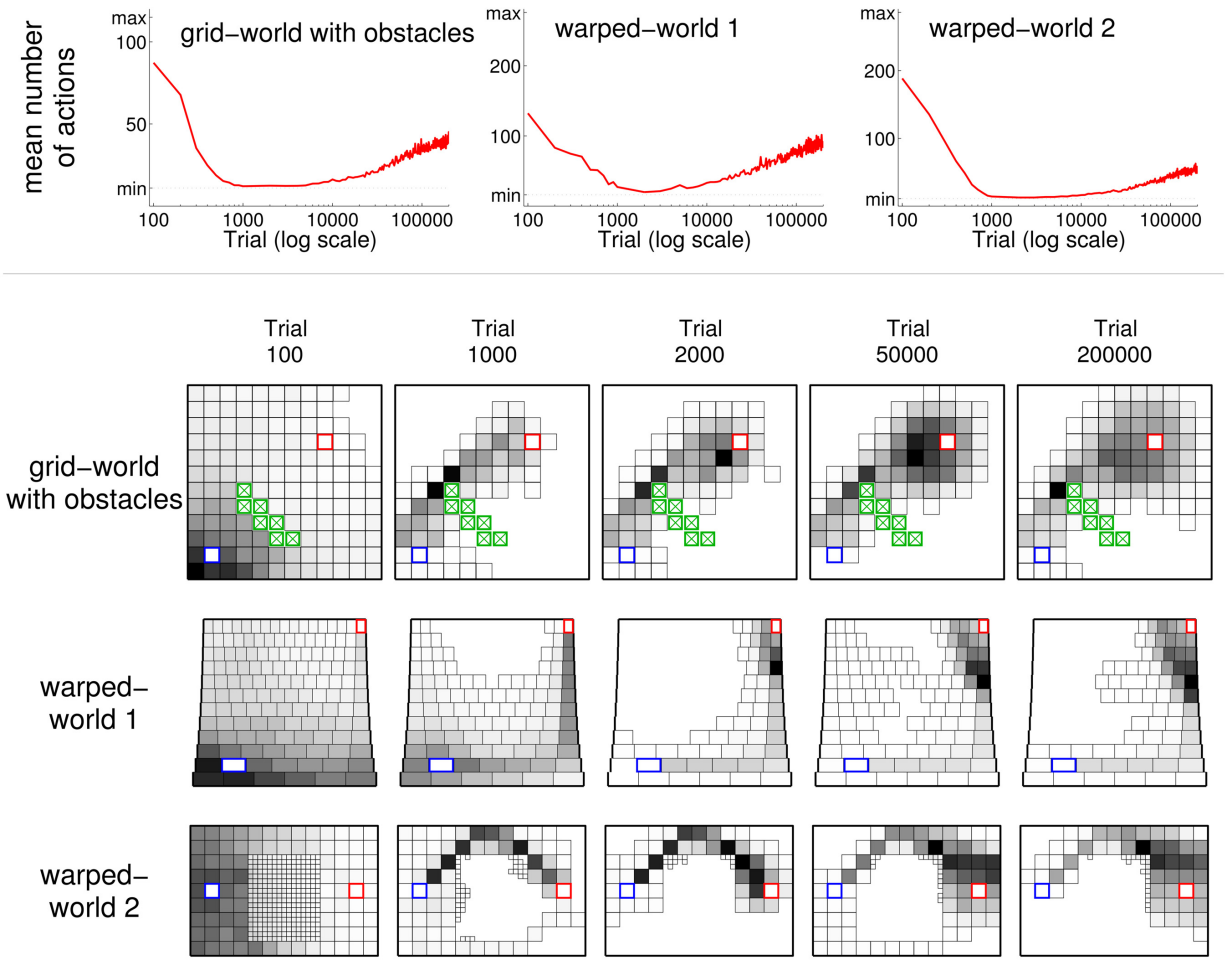
**Illustration of behavior for learning agents in the grid-world with obstacles and warped-worlds 1 and 2 using learning rule ncMC(0.7)**. (See Figure 1 for schematics of environments). Line plots (top graphs) follow conventions of Figure 2. Density of visited states plots (rest of graphs) follow conventions of Figure 6 bottom.

The general pattern of behavioral development observed in agents using ncMC(0.7) acting within the grid-world holds for agents acting within other environments (see Figure 1) as well. The top row of Figure 7 graphs, in a manner similar to Figure 2, the mean number of actions taken at each sample trial for agents using ncMC(0.7) acting within the grid-world with obstacles (left), warped-world 1 (middle), and warped-world 2 (right). The rest of Figure 7 shows, in a manner similar to Figure 6, the density of states visited for the three environments at different test points for sample trials that achieved *s^o^*.

Agents using ncMC(0.7) in the grid-world with obstacles discovered behavior that used the minimal action sequence (i.e., the shortest trajectory, above the obstacles). Thus, ncMC(0.7) discovered the minimal action sequence even when some short-length trajectories (e.g., above and below the obstacles) are not easily reached from each other (which increases the likelihood of getting stuck in a local minimum).

Agents in warped-world 1 produced behavior that first travels east to the border of the world, and then north. Agents in warped-world 2 produced behavior that avoids the middle of the environment by traveling along the upper region of the environment. Figure 7 shows that, in the grid-world with obstacles and the two warped-worlds, the minimal action sequence is discovered and executed for a temporary but substantial period of time. Also, as with the grid-world, behaviors under the no-cost rules in the other worlds do not converge: with continued reinforcement for an extended amount of time, extraneous actions, beginning at states near the outcome, are executed.

If spatially indirect overall behavior were observed (e.g., moving east and then north in warped-world 1, or moving above the center in warped-world 2) but the underlying state and action representations were not known, one possible account of such behavior would be that the actions executed at certain locations are simply more costly than actions executed at other locations (e.g., if moving horizontally along the north edge of warped-world 1 was very costly, and moving through the center of warped-world 2 was very costly), and that the learning rule incorporates these explicit action costs. Our results demonstrate that spatially indirect behavior can also be accounted for with other mechanisms: learning rules that do not incorporate explicit action costs, such as no-cost rules, govern behavior, and the underlying state representation is nonuniform on a spatial level.

We note that the spatially nonuniform state representation also allows for spatially indirect behavior to be accounted for by a learning rule that incorporates temporal discounting of the positive numerical signal received upon achievement of the outcome but does not incorporate explicit action costs. Note also that we do not suggest that a spatially nonuniform state representation prohibits the use of learning rules that incorporate explicit actions costs. Rather, we demonstrate how similar behavior can be accounted for with different mechanisms.

## 4. Discussion

Most sensory outcomes can be achieved through many different action sequences of varying lengths. Animals discover, through interaction with the environment and no outside instruction, the minimal action sequence—the minimal number of actions that achieves an outcome (Thorndike, 1911). The discovery of the minimal action sequence is often accounted for with learning rules that focus on “how well was the outcome achieved?” by associating actions with a measure of behavior that is higher for actions that lead to achieving the outcome with a smaller total number of actions. In this type of account, learning is driven by a prediction error in this measure of behavior, and the minimal action sequence is “optimal” in that it is associated with the highest measure of behavior. Factors that influence this measure of behavior in many accounts include the delivery of positive numerical signal if the outcome is achieved (which addresses the question “was the outcome achieved?”) along with some combination of explicit negative numerical signals (“costs”) for each executed action and/or temporal discounting of the numerical signals, either of which addresses the question “how well was the outcome achieved?” (Sutton and Barto, 1998).

However, such an account may not apply to all situations in which the minimal action sequence is discovered. In particular, in the process of *action discovery* (Redgrave and Gurney, 2006; Redgrave et al., 2008, 2011, 2013; Stafford et al., 2012; Gurney et al., 2013), the minimal action sequence is thought to be discovered by learning mechanisms that focus on the simple evaluation of “was the outcome achieved?” and are driven by a prediction error in the outcome's occurrence. As discussed in Redgrave and Gurney (2006), biological reinforcement signals in action discovery may occur too quickly to evaluate an action sequence beyond an indication of the outcome's occurrence.

In this paper we demonstrate that *no-cost* learning rules, which focus on “was the outcome achieved?” and are more consistent with action discovery than previous accounts, can also discover and execute the minimal action sequence for a temporary yet substantial period of time (Figures 2, 7). Under the no-cost rules described in this paper, if the outcome is achieved during a trial, the tendency to execute every action that was executed en route to the outcome is increased, but at a rate that decreases with temporal distance from the outcome (see Figure 3). In no-cost rules, though, every action that leads to achievement of the outcome is associated with the same measure of behavior. In effect, no-cost rules develop behavior that is similar to behavior developed by rules that focus on “how well was the outcome achieved?” but no-cost rules focus on the simple evaluation of “was the outcome achieved?”

One limitation of no-cost rules as described in this paper is that behavior does not converge if reinforcement continues for an extended period of time (Figures 2, 4, 7). This limitation is also consistent with the process of action discovery (Redgrave and Gurney, 2006; Redgrave et al., 2008), which suggests that a separate process that predicts the outcome's occurrence attenuates reinforcement signals as the outcome becomes predictable. (We do not model this proposed process in this paper). If such attenuation were disrupted, e.g., due to disorders of prediction or reinforcement functions, extraneous actions would be developed under no-cost rules, first appearing in close proximity to the outcome (Figures 4–6).

Another limitation, which arises with all scenarios involving learning without external instruction, is that of scaling. The environments we use (Figure 1) comprise between 100 and 1000 states. It is likely that, as with the more common with-cost RL (Sutton and Barto, 1998) rules we use in this paper, the effectiveness of no-cost rules will decrease if the number of states increases by a very large factor. One area of future research is to augment no-cost rules with techniques used to increase the effectiveness of with-cost rules in very large state spaces. These techniques include the development of state abstractions and behavioral hierarchies (Sutton et al., 1999; Dietterich, 2000; Barto and Mahadevan, 2003; Ravindran and Barto, 2003; Mahadevan, 2010; Osentoski and Mahadevan, 2010; Barto et al., 2013a) which should be applicable, in principle, to the no-cost rules we use here. We expect any limitations from scaling of our no-cost rules to be similar to those of with-cost RL rules.

We also note that, despite a similarity in language, our framework is different from that described in Friston et al. (2012). The latter does not invoke notions of optimality or cost because the agent already represents “optimal” behavior (such as the minimal action sequence) as a probability distribution over hidden states that is learned from experience generated by an external supervisor. The agent acts to move from low-probability (“surprising”) states that it does not expect to inhabit to high-probability states. Behavior is described in terms of information theoretic measures rather than optimal control.

Below we discuss computational and biological issues related to no-cost rules in behavioral development.

### 4.1. Differential rate of reinforcement

In computational RL (Bertsekas and Tsitsiklis, 1996; Sutton and Barto, 1998), the tendency, *Q*(*s*, *a*), to select action *a* from state *s* is modified with learning rules that modify *Q*(*s*, *a*) toward some target value (often referred to as the *return*). In many RL-based accounts of human or animal behavior, that target value is a measure of behavior that is influenced by a positive numerical signal (if the outcome is achieved) and also some combination of explicit action costs (negative numerical signals) and/or temporal discounting of numerical signals. (In the with-cost rules described in this paper, there are explicit action costs but no temporal discounting). In many tasks and environments, that target value is higher for actions that lead to shorter action sequences and, thus, *Q*(*s*, *a*) converges to a higher value if it reliably results in achievement of the outcome with a shorter action sequence. In contrast, in the no-cost rules described in this paper, the target value used to modify *Q*(*s*, *a*) is influenced only by a positive numerical signal if the outcome is achieved; explicit action costs and/or temporal discounting of the signals do not influence the target value. Thus, for tasks similar to those described in this paper, *Q*(*s*, *a*) for all (*s*, *a*) pairs converge to the same target value when modified with no-cost rules (see Methods for more details).

Even though *Q*(*s*, *a*) for all (*s*, *a*) pairs converge to the same value in no-cost rules, the minimal action sequence is discovered and executed for a substantial amount of time with (Figures 2, 7). A crucial feature of no-cost rules that enables them to find the minimal action sequence is that, if the outcome is achieved, proximal actions (which are executed in close temporal distance to the outcome) are reinforced at a greater rate than distal actions (executed in greater temporal distance from the outcome). This has the effect of reinforcing actions that lead to shorter action sequences that achieve the outcome at a greater rate than actions that lead to longer action sequences (Figure 3), and reinforcing the minimal action sequence at a greater rate than all other behaviors. If an external observer were not aware of the mechanisms by which behavior is developed and noted the execution of the minimal action sequence, he might describe such behavior as optimal with respect to a measure of behavior that is influenced by a positive numerical signal upon achieving the outcome and also some combination of explicit action costs and/or temporal discounting of numerical signals.

While there are likely many behaviors in which learning mechanisms associate behavior with a measure that is influenced by explicit action costs and/or temporal discounting, the central nervous system has multiple learning and control schemes at its disposal (Milner et al., 1998; Yin et al., 2008). By modifying different *Q*(*s*, *a*) at different rates toward the same target value, as opposed to modifying different *Q*(*s*, *a*) toward different target values, no-cost rules are able to discover and execute the minimal action sequence (temporarily) through different mechanisms and with different types of information than with-cost rules.

The differential rate of reinforcement can be accomplished with a decaying eligibility trace (Pavlov, 1927; Sutton and Barto, 1981, 1998; Klopf, 1982; Wörgötter and Porr, 2005) in Monte Carlo (MC) rules, which deliver reinforcement signals only at the end of a trial (such as rule ncMC(0.7)) when the outcome is achieved (Sutton and Barto, 1998). In Lecture III of his famous account of conditioned reflexes (Pavlov, 1927), Ivan Pavlov discusses how the *trace* of a conditioned stimulus (CS) allows behavior in response to the CS to be modified by an unconditioned stimulus (US, which produces the reinforcement signal) that occurs at a later time, and how the effect of reinforcement is weaker as delay between CS and US increases. Eligibility traces play a prominent role in several computational models of brain function (such as Suri and Schultz, 1998; Wörgötter and Porr, 2005; Izhikevich, 2007; Vasilaki et al., 2009; Chersi et al., 2013) and are used to describe several experimental results (Markram et al., 1997; Bi and Poo, 2001; Pan et al., 2005). They may be implemented in the brain through persistent neural activity (Goldman-Rakic, 1995; Curtis and Lee, 2010) or, as has been suggested in some modeling studies (Houk et al., 1995; Suri and Schultz, 1998), intracellular processes.

It is unclear if the influence of eligibility traces can extend to actions executed many time steps before the outcome in biological systems. However, the “bootstrapping” nature of temporal difference (TD) learning rules (Sutton, 1988; Sutton and Barto, 1998), in which intermediate states that predict a reinforcing event themselves become reinforcing, enables actions that are executed many time steps before the outcome to be reinforced. This paper demonstrates that TD rules also, in effect, reinforce actions proximal to the outcome at a faster rate than actions distal to the outcome. Thus, no-cost TD rules (such as ncTD(0)) can also discover and execute the minimal action sequence for a substantial period of time, even without eligibility traces. Recent experimental results (Wassum et al., 2012) demonstrate that dopamine (DA) release (thought to communicate reinforcement signals, Wickens et al., 2003, also discussed later in the Discussion) is propagated from proximal to distal actions in rats engaged in a operant conditioning task that requires a sequence of two separate actions in order to achieve an outcome.

Of course, the differential rate of reinforcement on which no-cost rules rely is not restricted to no-cost rules. MC rules and TD rules using with-cost measures with or without eligibility traces can easily be implemented (Bertsekas and Tsitsiklis, 1996; Sutton and Barto, 1998). The no-cost rules described in this paper allows us to more clearly demonstrate the functional mechanisms by which differential rates of reinforcement help shape behavioral development.

### 4.2. Dopamine activity

In order for behavior developed using no-cost rules to converge with extended experience, a separate process must attenuate reinforcement signals. Reinforcement signals in the brain are thought to be communicated by phasic DA neuron activity (henceforth referred to simply as *DA activity*). Experimental studies (Ljungberg et al., 1992; Schultz et al., 1993, 1997; Horvitz, 2000; Redgrave et al., 2011; Schultz, 2012) have shown that sensory-evoked DA activity attenuates with repeated presentations of the sensory stimulus. If mechanisms similar to no-cost rules participate in behavioral development, such participation provides a functional-level teleological explanation for why DA activity attenuation occurs: DA activity that is not attenuated by a separate process would result in prolonged reinforcement and consequential degradation of performance.

This interpretation is different than that in which DA activity is accounted for solely by the learning rule, i.e., in which the rule accounts for both an increase in DA activity (reinforcement) and its subsequent attenuation (Houk et al., 1995; Schultz et al., 1997). In this case, if the outcome can be achieved in many ways, it is necessary that the target value toward which *Q*(*s*, *a*) is modified represents a measure of behavior that is higher for actions that achieve the outcome in some “better” way than other actions that achieve the outcome (such as the with-cost measures described in the Methods). Otherwise, extraneous actions will occur. Most studies describing DA activity in such terms use fairly simple tasks (e.g., the outcome is biologically rewarding and is dependent on only one or two actions) to investigate how DA activity propagates from the outcome to otherwise neutral stimuli or actions that precede the outcome (Schultz et al., 1997; Schultz, 2012; Wassum et al., 2012) rather than how redundancy is resolved.

The putative separate process of attenuating DA activity if no-cost rules are used must rely on newly acquired knowledge, such as an internal prediction model of the outcome's occurrence, of the agent in relation to the task. In a critique of the role of DA activity and description of the process of action discovery (Redgrave and Gurney, 2006; Redgrave et al., 2008, 2011, 2013), Redgrave, Gurney, and colleagues suggest that short-latency (< 100 ms after a stimulus) DA activity indicates that something unexpected has happened (the outcome), but not its biological rewarding or reward-predicting properties (reward-related information may be communicated by longer latency DA activity). Thus, measures of behavior that represent “how well was the outcome achieved?” and that can be used to rank one action that achieves an outcome as better than another action that achieves the outcome may not be represented in action discovery. In this critique, the learning rule was not explicitly given, and it was proposed that a separate process learns to predict that the outcome will occur and attenuates DA activity accordingly. The no-cost rules described in this paper are consistent with the process of action discovery in that they focus on “was the outcome achieved?” as opposed to “how well was the outcome achieved?” but they do rely on the proposed separate process of attenuating DA activity based on a prediction of the outcome's occurrence. The separate process has been incorporated in a recent model which also investigates computational mechanisms consistent with action discovery (Bolado-Gomez and Gurney, 2013).

### 4.3. Further computational considerations

Behavior can result from functionality (such as reinforcement and attenuation of reinforcement) mediated by one process (as in with-cost rules), but that functionality can also be distributed across multiple processes (as in no-cost rules). Experimental studies describe how the development and execution of observed behavior in biological systems may be distributed across different learning and control processes (Dickinson, 1985; Milner et al., 1998; Packard and Knowlton, 2002; Pasupathy and Miller, 2005; Yin et al., 2008; Balleine et al., 2009; Balleine and O'Dohrety, 2010; Redgrave et al., 2010). Conceptual and computational accounts, inspired in part by these studies, demonstrate the functional advantages of such distribution (Kawato, 1990; Rosenstein and Barto, 2004; Daw et al., 2005; Haruno and Kawato, 2006; Samejima and Doya, 2007; Bissmarck et al., 2008; Shah and Barto, 2009; Ashby et al., 2010; Shah et al., 2013). Within the context of the work presented in this paper, no-cost rules reinforce actions that achieve the outcome but do not attenuate reinforcement. Separate prediction mechanisms (which we do not model here) would attenuate reinforcement signals and thus prevent the development of extraneous actions. In addition, other mechanisms may be trained by such behavior and then dominate control in a way that is specialized for executing, but not developing, behavior (computational examples of this are described in Shah, 2008 and Ashby et al., 2007).

Different learning and control mechanisms may have different advantages and disadvantages. For example, the with-cost rules described in this paper associate a different measure of behavior to different actions that lead to action sequences of different lengths in achieving the outcome. This is accomplished by taking into account explicit action costs—a negative numerical signal (the “cost”) that accompanies each executed action. Such a rule has advantages in that it is flexible: it is designed to find behavior that maximizes the measure of behavior given an arbitrary mapping from states and actions to positive and negative numerical signals, not just one in which the minimal action sequence is associated with the highest measure of behavior. For example, suppose that an outcome could be achieved by executing two particular actions, each of which incurs a cost of −1, or by executing three other actions, each of which incur a “cost” of 0. A learning rule that takes explicit action costs into account would learn to execute the latter sequence of three actions instead of the former sequence of two actions. However, this flexibility comes with a price on a computational and representational level: in such learning rules, resources must be devoted to represent every action's cost and incorporate those costs into learning signals. Such flexibility may not be necessary for many types of behavioral development, such as developing the minimal action sequence in action discovery, and it may be advantageous to use mechanisms that are less flexible but also are less expensive.

As discussed in the Introduction and Methods, another mechanism by which to associate actions that result in achieving the outcome with a shorter action sequence with a higher measure of behavior is to temporally discount the positive numerical signal received upon achieving the outcome (Sutton and Barto, 1998). This mechanism is less expensive (and less flexible) than incorporating explicit action costs, but, depending on the specific learning rule, it may be more expensive than no-cost rules. Consider the case of MC rules, which modify *Q*(*s*, *a*) for (*s*, *a*) visited during a trial only at the end of the trial. The computational steps executed during each trial for MC rules are different in temporal discounting of the positive numerical signal (*r_o_*) upon achieving the outcome vs. no-cost rules. In both rules, a variable *z*(*t*) is updated at each time step *t*. In rules that temporally discount *r_o_*, *z* is initialized to 1 and is then multiplied by γ (the temporal discount factor) at each time step (*z*(*t* + 1) ← *z*(*t*) γ). Similarly, in no-cost rules, *z* is initialized to α and is then multiplied by λ at each time step (*z*(*t* + 1) ← *z*(*t*) λ). However, when the end of the trial occurs and *Q*(*s*, *a*) for each visited (*s*, *a*) are modified, there is a difference. The temporal discount of *r_o_* rule computes three quantities before modifying *Q*(*s_t_*, *a_t_*): (i) *z*(*t*) *r_o_*, (ii) *z*(*t*) *r_o_* − Q(*s_t_*, *a_t_*), (iii) α[*z*(*t*) *r_o_* − *Q*(*s_t_*, *a_t_*)]. In contrast, the no-cost rule computes two quantities: (i) *r_o_* − *Q*(*s_t_*, *a_t_*), (ii) *z*(*t*)[*r_o_* − *Q*(*s_t_*, *a_t_*)]. Furthermore, although this analysis includes an update of *z*(*t*) at every time step in no-cost rules, such an update is independent of the specific task being accomplished in no-cost rules. Therefore, a sequence of these variables can be hard-wired in memory for use in any task rather than recalculated for every task. In rules that temporally discount *r_o_*, on the other hand, the multiplication involves a task-dependent variable (*r_o_*), so such a sequence must be calculated for every task and cannot be stored in memory for use in other tasks.

In conventional (von-Neumann style) computing hardware, *in silico*, these considerations are of little consequence. First, the hardware contains a general purpose processing unit, where each class of arithmetic operation (add, subtract, multiply, divide) is implemented only once; there is no dedicated hardware for particular instances of an arithmetic operation in a particular algorithm. Second, the “data” stored in memory [for each (*s*, *a*)] is separated from the arithmetic operations which operate on them. Thus, there is no extra hardware cost for rules that temporally discount the numerical signal upon achieving the outcome because those and no-cost rules use *common* arithmetic processing hardware, and have similar data/memory requirements. The former differs only in that it makes use of the arithmetic hardware more often.

In contrast, in the brain, while arbitrary arithmetic operations may be performed in neurons (Koch et al., 1983; Mel et al., 1998; London and Hausser, 2005), the neural substrate for each computation is usually *specific* to that computation, being embodied in a set of brain structures or nuclei. Moreover, processing (supported by trans-membrane currents in the neuron) is distributed across the “memory/data” (stored in synaptic weights) in a massively parallel way; there is no common processing unit bottleneck operating iteratively on a single data stream. Thus, in terms of neural circuits, the no-cost rules may be implemented using less “neural hardware” due to their fewer required arithmetic operations. This may be a significant factor for the biology implementing the different types of rules. The no-cost rules would require calculation of a signal to terminate training at the best point in time (see Results and Figure 2), but this is a single operation, common to all state-action pairs and therefore does not require a massively parallel computation.

Finally, recall that temporal discount of the positive numerical signal upon achieving the outcome in computational accounts is inspired by experimental studies in animal learning and behavioral economics (Samuelson, 1937; Chung, 1965; Logan, 1965; Green and Myerson, 2004). Temporal discount in most RL accounts are of an exponential form, which is relatively simple to implement mathematically in both MC and TD rules (Sutton and Barto, 1998). However, such a simple form may not govern animal behavior. An exponential temporal discount function exhibits a property sometimes referred to as *dynamic consistency* (Strotz, 1955; Thaler, 1981): if the relative difference in delay and magnitude between two rewarding outcomes is such that one is preferred (for example, preferring 1 apple today instead of 2 apples tomorrow), that preference is preserved even after a constant delay is added to both outcomes (preferring 1 apple in 365 days instead of 2 apples in 366 days). However, animal behavior exhibits dynamic inconsistency: 1 apple today might be preferred over 2 apples tomorrow, but that preference is reversed after a delay of a year is added, e.g., 2 apples after 366 days is preferred over 1 apple in 365 days, even though the relative delay between the two choices (1 day) is the same in both scenarios (Thaler, 1981; Myerson and Green, 1995, 1996). Other forms of temporal discount, such as hyperbolic functions or magnitude-dependent exponential functions, better explain animal behavior (Myerson and Green, 1995, 1996). Behavior described by hyperbolic temporal discounting may also be generated by the combination of different mechanisms that use different exponential temporal discounting (Kurth-Nelson and Redish, 2009). Also, different types of behavior may be governed by different forms of temporal discount (Green and Myerson, 2004). Thus, implementation of temporal discounting in animals may be more expensive on a computational and representational level than is usually assumed.

## 5. Concluding remarks

We have shown that no-cost rules, which focus on the simple evaluation of “was the outcome achieved?” and associate every action that leads to achievement of the outcome with the same measure of behavior, can be used to discover the minimal number of actions that achieves an outcome. Unlike previous accounts, which focus on “how well was the outcome achieved?” and associate actions with a higher measure of behavior if they lead to achieving the outcome with a smaller total number of actions, the no-cost rules we describe in this paper are consistent with the process of action discovery (Redgrave and Gurney, 2006; Redgrave et al., 2008, 2011, 2013; Gurney et al., 2013; Stafford et al., 2012). Although no-cost rules on their own will lead to behavior that includes extraneous actions if reinforcement were continued for an extended period of time, they can be used to find the minimal action sequence if they are part of a distributed system in which other processes attenuate reinforcement as the outcome's occurrence becomes predictable (Redgrave and Gurney, 2006; Redgrave et al., 2008; Gurney et al., 2013). No-cost rules are an account of behavioral development that uses different mechanisms and relies on different types of information than previous accounts of similar behavior.

## Author contributions

Ashvin Shah and Kevin N. Gurney conceived of the ideas and wrote the paper. Ashvin Shah developed and ran simulations and conducted analyses using Matlab (R2011) (Mathworks, Natick, MA, USA).

## Funding

We are grateful for financial support from the European Union's Seventh Framework Programme grant FP7-ICT-IP-231722 (“IM-CLeVeR: Intrinsically Motivated Cumulative Learning Versatile Robots”) and the United Kingdom's Engineering and Physical Sciences Research Council (EPSRC) “Green Brain” project EP/J019534/1.

### Conflict of interest statement

The authors declare that the research was conducted in the absence of any commercial or financial relationships that could be construed as a potential conflict of interest.
