# Supplementary material for: Finding minimal action sequences with a simple evaluation of actions
Source: Front Comput Neurosci. 2014 Nov 28;8:151. doi: 10.3389/fncom.2014.00151 (PMC4247113; doi:10.3389/fncom.2014.00151)
Supplement: Supplementary file 1 [file DataSheet1.PDF]

Frontiers Computational Neuroscience

Original Research Article
20148 November 2014
10.3389/fncom.2014.00151

Supplementary Material: Finding minimal action sequences with a simple evaluation of actions

Ashvin Shah [1,*], **Kevin N. Gurney** [1]

[1] *Department of Psychology, The University of Sheffield, Western Bank, Sheffield, S10 2TP, United Kingdom*

Correspondence*:
Ashvin Shah
Department of Psychology, The University of Sheffield, Western Bank, Sheffield, S10 2TP, United Kingdom, ashvin@gmail.com

1 SUPPLEMENTARY MATERIAL

This is supplementary material for the article: *Shah A and Gurney KN (2014). Finding minimal action sequences with a simple evaluation of actions. Frontiers in Computational Neuroscience 8:151 (doi: 10.3389/fncom.2014.00151).*

We provide here details regarding the effects of actions in the two warped-worlds (see Figure 1 (main text) bottom). In warped-world 1 (Figure 1 bottom left), only four cardinal actions are available (diagonal actions are not available). Actions are stochastic as follows (see Figure S1 left): Let l^n be the spatial length of side n of state s^0 (e.g., the center state in Figure S1 left). Let l^i be the spatial length of the border of state s^i that overlaps side n of s^0. Then, an action from s^0 will transport the agent to s^i a proportion l^i/l^n of the time. Because states are bordered by only one state to the east or west, actions east and west are deterministic. Because states are bordered by two states to the north or south, actions north and south are stochastic. Because the amount that the borders of states overlap is not consistent from state to state, the stochastic effects of vertical actions are state-dependent.

In warped-world 2, (Figure 1 (main text) bottom right), four cardinal and four diagonal actions are available. Also, "big" states represent a larger spatial area than "small" states: the area of a big state is 9 times the area of a small state. The effects of actions from big states to small states are as follows (Figure S1 middle): a cardinal action transports the agent to the neighboring small state that is aligned with the center of the big state. If a diagonal action from a big state does not transport the agent to another big state, then it will transport the agent to the small state bordering the small state that the neighboring cardinal action would have transported the agent to. For example, the diagonal action northeast would transport the agent to the small state that is to the north of the small state that is to the east of the center of the big state. The effects of actions from small states to big states are as follows: the agent is simply transported to the nearest big state (Figure S1 right).

2 SUPPLEMENTARY TABLES AND FIGURES

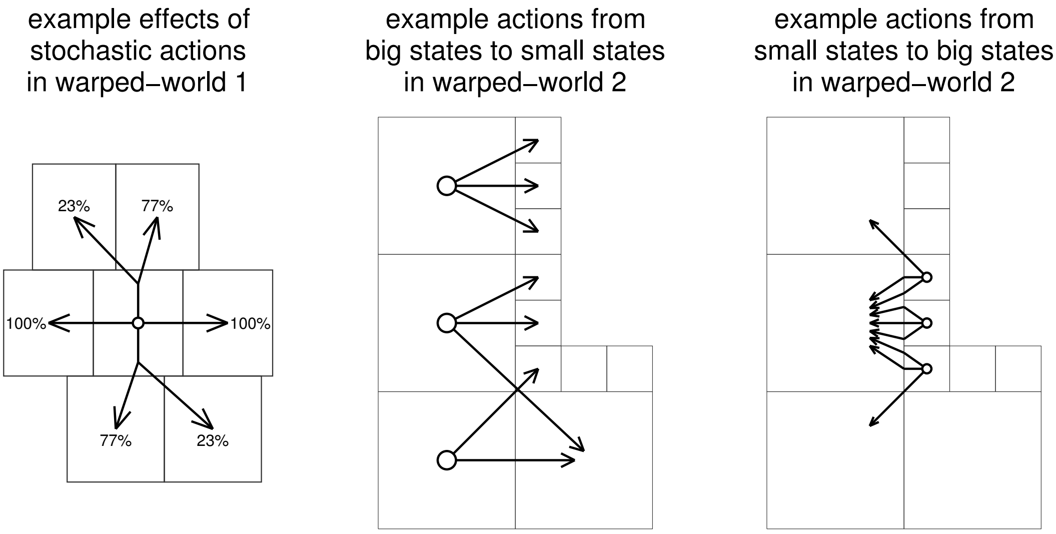

Supplementary Figure S1. Close up of states in the warped-worlds (see Figure 1 of the main text) to illustrate the effects of actions in the warped-worlds. Note that only a subset of actions are illustrated for warped-world 2 (middle and right panels).
